# Supplementary material for: Mechanical properties of lithic raw materials from Kazakhstan: Comparing chert, shale, and porphyry
Source: PLoS One. 2022 Apr 22;17(4):e0265640. doi: 10.1371/journal.pone.0265640 (PMC9033281; doi:10.1371/journal.pone.0265640)

PLOS ONE

Mechanical properties of lithic raw materials from Kazakhstan: comparing chert, shale, and porphyry

--Manuscript Draft--

**Manuscript Number:**	PONE-D-21-32866
**Article Type:**	Research Article
**Full Title:**	Mechanical properties of lithic raw materials from Kazakhstan: comparing chert, shale, and porphyry
**Short Title:**	Mechanical properties of chert, shale, and porphyry from Kazakhstan
**Corresponding Author:**	Abay Namen<br>Eberhard Karls Universitat Tubingen Philosophische Fakultat<br>Tübingen, GERMANY
**Keywords:**	Palaeolithic; mechanical properties; elasticity; hardness; fracture resistance.
**Abstract:**	The study of lithic raw material quality has become one of the major interpretive tools to investigate the raw material selection behaviour and its influence to the knapping technology. In order to make objective assessments of raw material quality, their mechanical properties (e.g., fracture resistance, hardness, modulus of elasticity) should be measured. However, such comprehensive investigations are lacking for the Palaeolithic of Kazakhstan. In this work, we investigate geological and archaeological lithic raw material samples of chert, porphyry, and shale collected from the Inner Asian Mountain Corridor (henceforth IAMC). Selected samples of aforementioned rocks were tested by means of Vickers and Knoop indentation methods to determine one aspect of their mechanical properties: their indentation fracture resistance (a value closely related to fracture toughness). These tests were complemented by traditional petrographic studies to characterise the mineralogical composition and evaluate the level of impurities that could have potentially affected the mechanical properties. The results show that materials, such as porphyry, previously thought to be of lower quality due to the anisotropic composition and coarse feldspar and quartz phenocrysts embedded in a silica rich matrix, possess fracture toughness values that can be compared to those of chert. Thus, it appears that different raw materials cannot be distinguished from the point of view of indentation fracture resistance, calling for detailed supplementary analyses of different fracture properties. This work also offers first insight into the quality of archaeological porphyry that was utilised as a primary raw material at various Middle and Upper Palaeolithic sites in the IAMC.
**Order of Authors:**	Abay Namen
	Radu Iovita
	Klaus G. Nickel
	Aristeidis Varis
	Zhaken Taimagambetov
	Patrick Schmidt
**Additional Information:**	
**Question**	**Response**
**Financial Disclosure**<br><br>Enter a financial disclosure statement that describes the sources of funding for the work included in this submission. Review the submission guidelines for detailed requirements. View published research articles from *PLOS ONE* for specific	This project has received funding from the European Research Council (ERC) under the European Union’s Horizon 2020 research and innovation programme (grant agreement n˚714842; PALAEOSILKROAD project).

Powered by Editorial Manager® and ProduXion Manager® from Aries Systems Corporation

examples.<br><br>This statement is required for submission and **will appear in the published article** if the submission is accepted. Please make sure it is accurate.<br><br>**Unfunded studies**<br>Enter: *The author(s) received no specific funding for this work.*<br><br>**Funded studies**<br>Enter a statement with the following details:<br>• Initials of the authors who received each award<br>• Grant numbers awarded to each author<br>• The full name of each funder<br>• URL of each funder website<br>• Did the sponsors or funders play any role in the study design, data collection and analysis, decision to publish, or preparation of the manuscript?<br>• **NO** - Include this sentence at the end of your statement: *The funders had no role in study design, data collection and analysis, decision to publish, or preparation of the manuscript.*<br>• **YES** - Specify the role(s) played.<br><br>* typeset	
**Competing Interests**<br><br>Use the instructions below to enter a competing interest statement for this submission. On behalf of all authors, disclose any competing interests that could be perceived to bias this work—acknowledging all financial support and any other relevant financial or non-financial competing interests.<br><br>This statement is required for submission and **will appear in the published article** if the submission is accepted. Please make sure it is accurate and that any funding sources listed in your Funding Information later in the submission form are also declared in your Financial Disclosure statement.	The authors have declared that no competing interests exist.

Powered by Editorial Manager® and ProduXion Manager® from Aries Systems Corporation

View published research articles from *PLOS ONE* for specific examples.<br><br>**NO authors have competing interests**<br><br>Enter: *The authors have declared that no competing interests exist.*<br><br>**Authors with competing interests**<br><br>Enter competing interest details beginning with this statement:<br><br>*I have read the journal's policy and the authors of this manuscript have the following competing interests: [insert competing interests here]*<br><br>* typeset	
**Ethics Statement**<br><br>Enter an ethics statement for this submission. This statement is required if the study involved:<br><br>• Human participants<br>• Human specimens or tissue<br>• Vertebrate animals or cephalopods<br>• Vertebrate embryos or tissues<br>• Field research<br><br>Write "N/A" if the submission does not require an ethics statement.<br><br>General guidance is provided below. Consult the submission guidelines for detailed instructions. **Make sure that all information entered here is included in the Methods section of the manuscript.**	The PALAEOSILKROAD project conducted all field research under license No. 15008746 (12.05.2015) of the National Museum of the Republic of Kazakhstan based on the collaboration protocol between the Eberhard-Karls University of Tübingen and the National Museum.

Powered by Editorial Manager® and ProduXion Manager® from Aries Systems Corporation

**Format for specific study types**<br><br>**Human Subject Research (involving human participants and/or tissue)**<br>• Give the name of the institutional review board or ethics committee that approved the study<br>• Include the approval number and/or a statement indicating approval of this research<br>• Indicate the form of consent obtained (written/oral) or the reason that consent was not obtained (e.g. the data were analyzed anonymously)<br><br>**Animal Research (involving vertebrate animals, embryos or tissues)**<br>• Provide the name of the Institutional Animal Care and Use Committee (IACUC) or other relevant ethics board that reviewed the study protocol, and indicate whether they approved this research or granted a formal waiver of ethical approval<br>• Include an approval number if one was obtained<br>• If the study involved *non-human primates*, add *additional details* about animal welfare and steps taken to ameliorate suffering<br>• If anesthesia, euthanasia, or any kind of animal sacrifice is part of the study, include briefly which substances and/or methods were applied<br><br>**Field Research**<br><br>Include the following details if this study involves the collection of plant, animal, or other materials from a natural setting:<br>• Field permit number<br>• Name of the institution or relevant body that granted permission	
**Data Availability**<br><br>Authors are required to make all data underlying the findings described fully available, without restriction, and from the time of publication. PLOS allows rare exceptions to address legal and ethical concerns. See the PLOS Data Policy and FAQ for detailed information.	Yes - all data are fully available without restriction

A Data Availability Statement describing where the data can be found is required at submission. Your answers to this question constitute the Data Availability Statement and **will be published in the article**, if accepted.<br><br>**Important:** Stating 'data available on request from the author' is not sufficient. If your data are only available upon request, select 'No' for the first question and explain your exceptional situation in the text box.<br><br>Do the authors confirm that all data underlying the findings described in their manuscript are fully available without restriction?	
**Describe where the data may be found in full sentences. If you are copying our sample text, replace any instances of XXX with the appropriate details.**<br><br>• If the data are **held or will be held in a public repository**, include URLs, accession numbers or DOIs. If this information will only be available after acceptance, indicate this by ticking the box below. For example: *All XXX files are available from the XXX database (accession number(s) XXX, XXX.).*<br>• If the data are all contained **within the manuscript and/or Supporting Information files**, enter the following: *All relevant data are within the manuscript and its Supporting Information files.*<br>• If neither of these applies but you are able to provide **details of access elsewhere**, with or without limitations, please do so. For example:<br><br>*Data cannot be shared publicly because of [XXX]. Data are available from the XXX Institutional Data Access / Ethics Committee (contact via XXX) for researchers who meet the criteria for access to confidential data.*<br><br>*The data underlying the results presented in the study are available from (include the name of the third party*	All relevant data are within the manuscript.

and contact information or URL).<br>• This text is appropriate if the data are owned by a third party and authors do not have permission to share the data.<br><br>* typeset	
Additional data availability information:	

Powered by Editorial Manager® and ProduXion Manager® from Aries Systems Corporation

Manuscript

Click here to access/download;Manuscript;namen_etal.2021-mechprop_final.docx

Mechanical properties of lithic raw materials from Kazakhstan: comparing chert, shale, and porphyry

Abay Namen[1,2], Radu Iovita[1,7], Klaus G. Nickel[3,4], Aristeidis Varis[1,5], Zhaken Taimagambetov[2,6], Patrick Schmidt[1,3]

[1] Department of Early Prehistory and Quaternary Ecology, University of Tübingen, Tübingen 72070, Germany

[2] Department of Archaeology, Ethnology and Museology, Faculty of History, Archaeology and Ethnology, Al Farabi Kazakh National University, Almaty 050040, Kazakhstan

[3] Department of Geosciences, Applied Mineralogy, Eberhard Karls University of Tübingen, Tübingen 72070, Germany

[4] Competence Center Archaeometry – Baden-Württemberg, Eberhard Karls University of Tübingen, Wilhelmstraße 56, Tübingen 72074, Germany

[5] Institute of Archaeological Sciences, Eberhard Karls University of Tübingen, Tübingen 72070, Germany

[6] National Museum of the Republic of Kazakhstan, Nur-Sultan 010000, Kazakhstan

[7] Center for the Study of Human Origins, Department of Anthropology, New York University, New York 10003, USA

Abstract

The study of lithic raw material quality has become one of the major interpretive tools to investigate the raw material selection behaviour and its influence to the knapping technology. In order to make objective assessments of raw material quality, their mechanical properties (e.g., fracture resistance, hardness, modulus of elasticity) should be measured. However, such

comprehensive investigations are lacking for the Palaeolithic of Kazakhstan. In this work, we investigate geological and archaeological lithic raw material samples of chert, porphyry, and shale collected from the Inner Asian Mountain Corridor (henceforth IAMC). Selected samples of aforementioned rocks were tested by means of Vickers and Knoop indentation methods to determine one aspect of their mechanical properties: their indentation fracture resistance (a value closely related to fracture toughness). These tests were complemented by traditional petrographic studies to characterise the mineralogical composition and evaluate the level of impurities that could have potentially affected the mechanical properties. The results show that materials, such as porphyry, previously thought to be of lower quality due to the anisotropic composition and coarse feldspar and quartz phenocrysts embedded in a silica rich matrix, possess fracture toughness values that can be compared to those of chert. Thus, it appears that different raw materials cannot be distinguished from the point of view of indentation fracture resistance, calling for detailed supplementary analyses of different fracture properties. This work also offers first insight into the quality of archaeological porphyry that was utilised as a primary raw material at various Middle and Upper Palaeolithic sites in the IAMC.

Key words: Palaeolithic; mechanical properties; elasticity; hardness; fracture resistance.

Corresponding author: Abay Namen (abay.namen@uni-tuebingen.de) at Hölderlingstr. 12, Tübingen 72076, Germany

1 Introduction

In recent years, the study of differences of lithic raw materials for the production of stone tools has received much attention because different types of rocks selected to produce lithics influence the technology and type of tools manufactured [1–6]. It also helps to understand the way in which rock attributes affect reduction sequences and edge-wear properties of tools [2,4,7]. Sedimentary rocks such as chert, flint, silicified shale, and other silica rich rocks were commonly used due to their predictable fracturing properties and good knapping qualities. Raw

material quality is commonly related with the mineralogical structure (e.g., grain size and shape) and purity of a given material. In most cases, raw materials that have isotropic mechanical properties are often considered to be of higher quality for tool making. Microstructural characteristics are also thought to potentially affect the size and/or shape of a final knapped product [2]. This is supported by replicative experiments conducted by contemporary knappers which have demonstrated that higher quality raw material has a direct influence on the manufacturing process [8–15].

Some scholars argue that a good raw material that is suitable for knapping should be brittle, elastic, and isotropic [9,10]. In the past, only few semi-quantitative and quantitative studies of the mechanical properties have been carried out, and these date to the mid-20th century. Goodman's [1944] experimental studies on this subject have been one of the first to study the hardness, toughness, and density of archaeological stone tools. Every stone tool can be seen as a unique material that has different raw material structure, morphology, and composition. The study by Moník and Hadraba [2016], attempted to answer the question of whether differences in raw material procurement may have been driven by the mechanical properties of selected materials or not. Studies conducted by Lerner et al. [2007] suggest that the physical properties of lithic raw materials have direct implications for use-wear accrual rates, meaning that roughness is linked to the rate of wear on stone tools. However, similar works in mechanical characterization of raw materials from archaeological contexts are found in limited quantities. Some of the available studies concern mineralogical, chemical and crystallographic transformations [19], as well as few detailed studies were dedicated to determine the thermal evolution of fracture mechanics [20–23]. The importance of the studies of mechanical properties become more pronounced due to the growing body of evidence that indicates deliberate alteration of lithic raw material properties by hominins to increase the knapping qualities of rocks which has implications for the evolution of human cognition [20,22–26].

The current work is based on a systematic investigation of archaeological stone tools and geological samples of raw materials to determine the mechanical properties from different Upper Palaeolithic sites of Kazakhstan **(Fig. 1)**. The primary objectives are (1) to test geological and archaeological samples to determine their mechanical properties, and (2) to preliminarily assess how these properties affect the knapping technology. Previous studies have been primarily concentrated on silica materials such as chert and silcrete [18,20,23], but other types of sedimentary and volcanic rocks found in archaeological contexts have received less attention. Here, we attempt to correct this imbalance by testing both sedimentary and volcanic rocks used by prehistoric societies.

In this paper, we analyse chert, shale, and porphyry using indentation testing to investigate three mechanical properties: fracture resistance, elasticity (also known as Young's modulus), and hardness **(Table 1)**. Additionally, traditional petrographic analysis was carried out to assess the mineralogical composition, impurities within the matrix, and individual grain size of minerals that could potentially influence the fracture behaviour of the studied specimens.

2 Regional setting

The study areas of the current work are the piedmont and foothill zones of the Qaratau, Ili Alatau, and the Altai-Tarbagatai mountain ranges located in the southern, south-eastern, and eastern Kazakhstan, respectively **(Fig. 1)**. It includes much of the Inner Asian Mountain Corridor (henceforth IAMC) [Frachetti, 2012]. The IAMC is a chain of mountain ranges in the centre of the Eurasian continent stretching approximately 2000 km and occupying most of the Central Asian countries, western China, and Mongolia. Although geologically different, the study areas share common geological formation characteristics, and topographic expressions originate from the tectonic activity, erosion, and other depositional processes. The mountain ranges under study are all affected by a fault system that separates into different sub ranges, i.e., the Greater and Lesser Qaratau.

Many Palaeolithic sites are found in the Qaratau range. Its structural and geological settings have been previously characterised by a number of geologists [28–31]. The range mainly consists of Neoproterozoic and Palaeozoic bedrocks, and several carbonate seamounts developed due to thermal subsidence of the newly formed crust. The major carbonate platform formed during the Famennian and early Pennsylvanian [31]. The formation of the carbonate platform affected the structure of the Qaratau range being mainly composed of limestone which creates a precondition to form caves, rockshelters and the formation of silica rich rocks within carbonate beds. Such environmental factors played an important role in the human occupation of the region.

Unlike the Qaratau range, the Ili Alatau mountain range (Kazakh portion of the northern Tian Shan) is characterised by steep slopes and the presence of glaciers at higher elevations [28]. The northern foothills enclose vast depression of the Ili and Dzungarian Alatau to the north-east. The mountain foothills are characterised by a blanket of different types of sediments, of which loess covers most of the area. The loess blanket known as the Central Asian piedmont that extends from the Pamir and Alai to the Ili Alatau was extensively studied and defined by Fitzsimmons et al. [2017].

These mountain ranges make up of a highly seasonal region with semi-arid and arid climatic conditions. The position of the mountain groups has played an important role in the early hominin dispersal across Asia [33–36], and possibly provided a refugium during the colder time in Pleistocene [37–39]. Already existing archaeological data indicate an extensive human activity, and earlier studies concerning the Palaeolithic of Kazakhstan had already revealed that this region has a potential to study the patterns of human behaviour and migration throughout the Central Asia [32,36,40–53].

The studies related to the raw materials of lithic assemblages remain limited. Recent work by Namen et al. [2020b] describes the raw material distribution in the piedmont zones of

Kazakhstan using the Centre for Russian and Central EurAsian Mineral Studies (CERCAMS) database and discusses the geographic use patterns of different raw materials. According to macroscopic observations of lithic assemblages, every stratified site has a distinctive type of raw material, probably outcropping locally in a close vicinity to the sites. Due to complex geological formations of the piedmont and foothill zones of the IAMC, this region offers a large amount of knappable raw materials. Therefore, the Palaeolithic sites across Kazakhstan has assemblages knapped on variety types of lithologies. Our work showcases these differences by comprehensive investigation of different rocks as well as offers a detailed insight into their mechanical properties. Thus, comparison between sites utilising different lithologies are not meaningful without understanding the limitations that each raw material imposes to the knapper.

Fig 1. Palaeolithic sites mentioned in the text and illustrated in relation to the major topography of the Inner Asian Mountain Corridor (Kazakh portion). 1) Yntaly. 2) Usiktas (surface site), 3) Valikhanova, 4) Kattasai, Uzbekistan, 5) Maibulaq, 6) Rahat 7) Ushbulaq. Data sources: Global Administrative areas (GADM) [55], vector and raster map data from Natural Earth (www.naturalearthdata.com) and Shuttle Radar Topography Mission (SRTM) Version 4 [56].

Table 1. Description of analysed samples.

Sample ID	Rock type	Location	Sample type	References
UBD-1-20*	Dark shale	Ushbulaq, East Kazakhstan	geological	Namen et al. [020b]
UBG-1-20*	Green shale	Ushbulaq, East Kazakhstan	geological	Namen et al. [2020b]
YNT-1-20*	Chert	Yntaly, South Kazakhstan	geological	Namen et al. [2020b]
MB-1-20*	Porphyry	Maibulaq, South Kazakhstan	archaeological	Fitzsimmons et al. [2017]
UT-22*	Chert	Usiktas, South Kazakhstan	archaeological	Alpysbaev [1979]
UT-144*	Chert	Usiktas, South Kazakhstan	archaeological	Alpysbaev [1979]
UB-526*	Dark shale	Ushbulaq, East Kazakhstan	archaeological	Anoikin et al. [2019]

UB-537*	Green shale	Ushbulaq, East Kazakhstan	archaeological	Anoikin et al. [2019]
UB-571	Green shale	Ushbulaq, East Kazakhstan	archaeological	Anoikin et al. [2019]
UB-532	Dark shale	Ushbulaq, East Kazakhstan	archaeological	Anoikin et al. [2019]
UB-492	Green shale	Ushbulaq, East Kazakhstan	archaeological	Anoikin et al. [2019]
UB-514	Dark shale	Ushbulaq, East Kazakhstan	archaeological	Anoikin et al. [2019]
UB-616	Limestone	Ushbulaq, East Kazakhstan	archaeological	Anoikin et al. [2019]
UT-48	Chert	Usiktas, South Kazakhstan	archaeological	Alpysbaev [1979]
UT-10	Chert	Usiktas, South Kazakhstan	archaeological	Alpysbaev [1979]
UT-181	Chert	Usiktas, South Kazakhstan	archaeological	Alpysbaev [1979]
UT-217	Chert	Usiktas, South Kazakhstan	archaeological	Alpysbaev [1979]

Localities shown in the table are illustrated on Figure 1. Samples marked with (*) were tested to determine their mechanical properties, the rest were petrographically characterised.

3 Materials and methods

3.1 Samples and sample preparation

For the current study, we have selected a total of 17 samples, 14 of them are archaeological lithics and the remaining 3 are geological raw materials **(Table 1)**. The geological samples were collected during the PSR project's field campaign conducted in 2018 and 2019 [54,57]. Primarily, chert and shale outcrops were surveyed and sampled because they were commonly used as lithic raw materials and produced stone tools in different archaeological sites in the piedmont and foothill zones of the IAMC [54]. The archaeological samples of lithics come from the stratified sites of Ushbulaq (Eastern Kazakhstan) [52,58], Maibulaq (Almaty region) [32,59,60] and a surface site at Usiktas (South Kazakhstan) [61]. Chert can be frequently found in stratified and surface sites throughout southern Kazakhstan, and shale is a major raw material of stone tool assemblages from Ushbulaq. Porphyry was commonly utilised in stratified sites at Maibulaq and Rahat [62]. We refer to porphyry as a rock of volcanic origin with crystals visible to the naked eye set in a fine grained matrix. The samples and their descriptions are summarized on **Table 1**. Location of the sites are shown in **Fig. 1**.

Fig 2. Illustration of archaeological (A-E) and geological (F-H) samples that undergone mechanical testing by means of indentation tests. The dotted lines are the impressions of the diamond indents.

3.2 The indentation tests

A total of 8 samples (5 archaeological and 3 geological) were tested to determine their mechanical properties. In order to conduct the analyses, plane-parallel plates measuring approximately 40 × 30 × 3 mm (thickness of each sample varies) were cut and diamond polished (see **Fig. 2**) on one side. They were then analysed by the Vickers and Knoop indention.

These indentations provide data on indentation fracture resistance (according to the method proposed by [63], Young's modulus (according to the method proposed by Ben Ghorbal et al. [65]) and hardness of each sample. The protocol for indentations was as follows. The load was set to 100 N (with a pre-load of 10 N), speed of indentation 1 mm/s and a hold time of 20 s. Both indenters produce either square-shaped or diamond-shaped elongated indentations. The size of each indentation is proportional to material hardness according to equation (1). Another consequence of the indentation of brittle materials with Vickers and Knoop indenters is the formation of half penny-shaped cracks that can be observed at the polished surface. Cracks apparently depart from all four corners of Vickers indentations and from the two acute angle corners of Knoop indentations. The length of the cracks forming from Vickers indentations are proportional to the value of indentation fracture resistance (a value closely related to fracture toughness (see [64]).

Indentation tests were performed using an Instron 4502 universal testing machine at the laboratory for Applied Mineralogy at Tübingen University's Geosciences department, Germany. Each sample was indented 20 times for each type of indenter to obtain statistically relevant data (see **Fig. 2**). The size of indentations and cracks was determined using images acquired with a HITACHI Tabletop scanning electron microscope (SEM) TM3030.

Data treatment was as follows. *Knoop hardness* was calculated by using the formula suggested by Ben Ghorbal et al. [65]. They proposed a way to calculate Knoop hardness that yields results equivalent to the Vickers hardness (equation 12 in Ben Ghorbal et al. [65]):

$$HK = \frac{P}{\frac{b'}{2} \cdot L'} \tag{1}$$

where,

P is the applied load in KgF;

b' is the short diagonal of the Knoop indent in mm;

L' is the long diagonal of the Knoop indent in mm.

Modulus of elasticity is normally obtained by measuring the potential of a material to deform upon applied stress. However, as bending tests lied outside of the possibilities for this study, we used Ben Ghorbal et al. [65]'s formula that relies on comparing the long and short diagonals of the Knoop indentations. The calculation was initially suggested by Marshall et al. [66], but further refined by Ben Ghorbal et al. [65]:

$$E = 0.417 \times HK \div \left(\frac{1}{7.11} - \frac{b'}{L'}\right) \tag{2}$$

where,

HK is the Knoop hardness in GPa;

b' is the short diagonal of the Knoop indent in µm;

L' is the long diagonal of the Knoop indent in µm.

Indentation fracture resistance, as defined by Danzer et al. [64], closely related to fracture toughness, a measure of a material's resistance to fracture propagation. We use Niihara et al. [63]'s formula to obtain a value for indentation fracture resistance *(K_{Ic})*, which uses the length of the surface cracks that develop from the four apical points of the Vickers indentation:

$$K_{Ic} = 0{,}067 H\sqrt{a}\ \left(\frac{E}{H}\right)^{0{,}4} \left(\frac{c}{\mathrm{a}}\right)^{-1{,}5} \tag{3}$$

where,

E is Young's modulus in MPa;

H is Vickers hardness in MPa (we used VH here, as calculated from Ben Ghorbal et al. [65], as the differences between HV and HK are negligible when using their calculations);

c is the length of the cracks in µm;

a is the diagonal of the Vickers indentation in µm.

For this calculation, we admitted that the Knoop hardness is equal to the Vickers hardness and use the value obtained by eq. (1). Because the size of some of our Vickers indentations could not be accurately determined because their edges flaked off during the experiments, we admitted a standard value of quartz that is equal to 11.65 GPa. Diagonals of those indentations with intact margins were measured.

3.3 Petrographic study

A total of 16 thin sections, consisting of 12 archaeological samples from surface collections and 4 geological reference samples were microscopically examined. We assessed potential impurities, mineralogical composition, the grain size of quartz grains and relative percentages of clasts and matrix (see **Table 4**). Traditional petrographic analysis was conducted at the laboratory for Geoarchaeology of the Institute for Archaeological Sciences (INA), University of Tübingen, Germany. The geological and archaeological (surface lithics) samples were prepared following a standard thin section preparation procedure. The thickness of the samples was reduced on a thin section grinder until the thickness of ca. 3 µm reached. Thin sections were analysed using a Zeiss petrographic microscope and photomicrographs were obtained using Axio camera coupled to the microscope. Identification and description of minerals were made under plane polarized light (PPL) and cross-polarized light (XPL) following the terminology outlined by Courty et al. [68] and Stoops [69].

Thin sections of samples tested for mechanical properties were analysed employing the Image J software to calculate the percentages of inclusions and matrix which were estimated using the point counting technique [70]. We aimed to identify these components to evaluate whether there is a relationship with mechanical properties or not.

4 Results

3 out of 8 samples yielded mechanical values because of the flaking off of all margins which made it impossible to measure indentation diagonals or crack lengths for the other samples. Thus, our results rely on few successful indentations and, therefore, the whole scheme must be regarded with some caution. Here, we present the results obtained from chert, shale, and porphyry as well as describe their petrographic features. Values of hardness, elastic modulus, and indentation fracture resistance are summarised below in **Tables 2 and 3** and graphically shown in **Fig. 3-5**. Photomicrographs of studied thin sections are shown in **Fig 6. and 7**.

Table 2. Hardness, elasticity, and fracture resistance values as calculated from the indentation tests.

**Sample ID**	**Rock type**	***HK***	***E***	K_{Ic}
UT-22	Chert	10.153	77	2.4
UB-537	Shale	4.392	153	3.01
MB-1-20	Porphyry	6.730	55	1.6

Note: the values of *HV* and *HK*, and *E* are given in GPa, and K_{Ic} is given in MPa $m^{1/2}$

Table 3. The values of hardness, elastic modulus, and fracture resistance as calculated from the Vickers and Knoop indentation.

**Sample name**	**Crack length of Vickers**		**Crack length of Knoop**		***HK***	***E***	K_{Ic}

	**diamond**		**diamond**				
	c [µm]	***a [µm]***	***L' [µm]***	***b' [µm]***	***[GPa]***	***[MPa]***	***[MPa m$^{1/2}$]***
**UT-22 (Chert)**	188	125	495	36	11.179	68724	**2.38**
**UT-22 (Chert)**	188	125	483	44	9.283	80041	**2.27**
**UT-22 (Chert)**	188	125	487	37	10.984	71581	**2.40**
**UT-22 (Chert)**	188	125	480	34	12.093	73124	**2.56**
**UT-22 (Chert)**	188	125	489	41	9.786	73606	**2.26**
**UT-22 (Chert)**	188	125	470	37	11.411	77487	**2.53**
**UT-22 (Chert)**	188	125	488	48	8.368	86450	**2.20**
**UT-22 (Chert)**	188	125	480	44	9.418	80573	**2.29**
**UT-22 (Chert)**			476	43	9.644	81872	
**UT-22 (Chert)**			483	44	9.363	79105	
**UB-537 (Shale)**	139	125	590	77	4.386	188498	**3.17**

**UB-537**	132	125	603	75	4.397	117266	**2.84**
**(Shale)**	122	125					
**UB-537**	136	125					
**(Shale)**	122	125					
**UB-537**	137	125					
**UB-537**	137	125					
**(Shale)**	156	125					
**MB-1-20** **(Porphyry)**	200	125	551	49	7.298	60380	**1.59**
**MB-1-20** **(Porphyry)**	177	125	601	53	6.162	50410	**1.61**
**MB-1-20** **(Porphyry)**	204	125					
**MB-1-20** **(Porphyry)**	182	125					
**MB-1-20** **(Porphyry)**	199	125					
**MB-1-20** **(Porphyry)**	195	125					
**MB-1-20** **(Porphyry)**	102	125					
**MB-1-20** **(Porphyry)**	182	125					
**MB-1-20** **(Porphyry)**	155	125					

**MB-1-20 (Porphyry)**	143	125					
**MB-1-20 (Porphyry)**	147	125					
**MB-1-20 (Porphyry)**	176	125					

Note that the diagonal value (*a*) was admitted from the standard quartz diagonal due to the flaking off of the edges. It was also admitted as a standard diagonal for the rest of the indented samples.

4.1 Indentation tests

Among the three different types of lithologies studied, chert (UT-22) shows the highest hardness value of over 10 GPa compared to 6.730 GPa of porphyry (MB-1-20) and 4.392 GPa of shale (UB-537). Based on these experiment results, hardness of shale is lowest as compared to the other two rocks.

Fig 3. The hardness values.

The modulus of elasticity, calculated using eq. (2), suggests that porphyry is the less stiff material with a mean value equal to 55 GPa. Whereas chert yielded 77 GPa and shale is the stiffest rock among studied lithologies with an E value of 153 GPa. Although porphyry is a volcanic rock that contains relatively large phenocrysts of quartz and feldspar, our results suggest that it is less stiff than the other two analysed samples.

Fig 4. The modulus of elasticity values.

The K_{Ic} values suggest that shale is most resistant to fracture with a mean value of 3.01 MPa $m^{1/2}$. The samples of chert and porphyry have a value equal to 2.4 and 1.6 MPa $m^{1/2}$,

respectively. These data may suggest that among three lithologies fracture propagates without consuming much energy in porphyry than in those of shale and chert. However, more detailed investigation of rock strength is required to determine whether lower K_{Ic} value equals to easier flake detachment.

Fig 5. The fracture resistance of studied samples.

4.2 Petrography

Table 3 summarizes the petrographic characteristics of the studied rock samples. Prior to petrographic analysis, samples were macroscopically identified as shale, chert, limestone, and porphyry.

During petrographic analysis, two types of shales were distinguished: calcareous shale (for instance UBG-1-20) mainly comprised of well-sorted, from subrounded to rounded crystals of calcite and quartz supported by a possibly calcareous matrix. Calcite grains within shale is of secondary origin which was recrystallised during later rock diagenesis. The second type is siliceous shale (UBD-1-20) that consists of subrounded grains of feldspar and quartz. Thin sections prepared from archaeological lithics from Ushbulaq (*n*=6) show similar petrographic characteristics as the geological samples with a varying degree of quartz, calcite, and feldspar inclusions with the exception of sample UB-616.

The thin sections of archaeological lithics from Usiktas (*n*=6) are entirely composed of length-fast chalcedony similar to the geological sample of YNT-1-20 (Yntaly chert). Microscopically, these samples have similar microstructure. However, we do not suggest that these lithic raw materials were transported from Yntaly, we only note their structural similarity. The presence of impurities and other minerals within chert samples are not observed. A thin section of porphyry from Maibulaq (MB-1-20) is comprised of coarse and angular grains of quartz and

feldspar supported by a siliceous matrix. An inclusion of xenoliths, possibly broken off from the magma chamber or conduit walls around the time of eruption was observed (**Fig. 6A**).

Fig 6. Photomicrographs of A) porphyry, B) shale, and C) chert under XPL (upper) and PPL (lower) lights that were tested by means of indentation.

Table 4. Basic petrographic description of archaeological and geological thin sections.

Sample ID	Rock type	Matrix	Description
MB-1-20	Porphyry (Rhyolite ?)	Siliceous	Coarse and angular grains of quartz, feldspar, and sericite, a product of hydrothermal alteration of feldspar, minerals embedded in a fine-grained siliceous matrix. Large xenolith is observed.
UBD-1-20	Dark shale	Calcareous	Fine-grained, rounded and subrounded grains of quartz and calcite are supported by a calcareous matrix.
UBG-1-20	Green shale	Siliceous	Moderately sorted, subrounded grains of quartz and feldspar, and fine-grained calcite minerals are supported by a siliceous matrix.
YNT-1-20	Chert	Siliceous	Entirely composed of length-fast chalcedony
UB-537	Dark shale	Siliceous	Rounded, microcrystalline quartz grains embedded in a siliceous matrix.
UB-492	Green shale	Siliceous	Subrounded grains of quartz and calcite supported by a siliceous matrix.
UT-48	Chert	Siliceous	Entirely composed of length-fast chalcedony.
UT-10	Chert	Siliceous	Entirely composed of length-fast chalcedony.
UT-181	Chert	Siliceous	Entirely composed of length-fast chalcedony.
UT-144	Chert	Siliceous	Entirely composed of length-fast chalcedony.
UT-22	Chert	Siliceous	Entirely composed of length-fast chalcedony.
UT-217	Chert	Siliceous	Entirely composed of length-fast chalcedony.

UB-571	Green shale	Siliceous	Primarily composed of well-rounded, well-sorted quartz grains, few inclusions of angular feldspar can be observed.
UB-532	Dark shale	Siliceous	Composed of primarily well-sorted calcite and few quartz minerals, several internal cracks are filled with calcite.
UB-514	Dark shale	Siliceous	Subrounded grains of clay sized quartz supported on a siliceous matrix.
UB-616	Silicified limestone (?)	Calcareous	Silicified limestone.

In addition, individual grain sizes of quartz, percentage of inclusions and matrix of indented samples (see **subchapter 4.1** and **Table 1**) has been measured and shown in **Table 4**. This is done to examine whether petrographic characteristics (grain size, number of inclusions and matrix) affects the fracture resistance of tested rocks. As expected, porphyry contains the largest grain size among other studied samples. These clasts are also visible to the naked eye. The geological samples of shale contain relatively large mineral grains with a mean size equal to 299.5 µm, whereas the grain size of archaeological lithic is much smaller and equals to 41.9 µm. Average grain size of chert could not be measured due to the cryptocrystalline nature of quartz grains in chalcedony.

Fig 7. Photomicrographs of A) chert, B) calcareous shale, C-D) and siliceous shale under XPL (upper) and PPL (lower) lights.

Table 5. Grain size of quartz minerals and percentage of inclusions and matrix. CC stands for cryptocrystalline grain size (<1 µm). Note: samples marked with (*) were tested to determine their mechanical properties.

Sample ID	Rock type	% inclusions	%matrix	Quartz		
				Min (µm)	Max (µm)	Mean (µm)

UBD-1-20	Dark shale	34.7	55.1	146.6	566.2	299.5
UBG-1-20	Green shale	10.2	89.8	77.2	740.8	386.9
MB-1-20*	Porphyry	32.9	60.6	409.5	2241.9	1125.4
UB-537*	Shale	55.1	61.3	25.1	89.3	41.9
UT-22*	Chert	CC	CC	CC	CC	CC

5 Discussion

This experimental work is one of the first to be implemented for the Palaeolithic of Kazakhstan. However, our discussion relies on few successful indentations (**Table 3**) and, therefore, the whole scheme must be regarded with some caution. Despite the small size of studied samples, this work offers insights into some mechanical properties of chert, shale, and porphyry that add to the knowledge of the knapping technology and stone tool assemblage formation. The results suggest that the raw materials considered as a lower quality material (porphyry) due to the presence of large phenocrysts, in fact, have mechanical properties (e.g., fracture resistance and modulus of elasticity) that can be compared to the materials that are considered to be of higher quality, i.e., chert and silcrete studied by Schmidt et al. [23].

5.1 Raw material quality

The studied mechanical properties allow us to have a preliminary discussion on raw material quality. Our results suggest that chert is the hardest material, harder than porphyry and shale. This is expected since it is entirely composed of length-fast chalcedony, which in turn is quartz (with an average hardness of ~12 GPa). On the other hand, shale has a lower hardness value in spite of its siliceous matrix and primarily quartz composition as seen under microscope. Although impurities within the shale have not been observed during our petrographic study, the lower overall hardness of the rock could be due the presence of clay impurities within the shale microstructure that was not detectable under 40x magnification [67]. The *E* values obtained from our experiments likewise show significant differences between chert, shale, and porphyry.

Even though chert is composed of chalcedony and shale consists primarily of microcrystalline quartz, the elasticity values of chert and shale differ greatly, too. On the other hand, the *E* values of porphyry are lower than those of chert and shale. In the analysis of mechanical properties of heat-treated silcrete, Schmidt et al. [2019] observed that silcretes with more (abundant) clasts generally had a strong loss of *E* values upon heat treatment compared to finer silcrete. This means that structural differences developed during the heat treatment process may affect the elasticity value [23]. This is also supported by our observations on the relationship of grain size and mechanical properties of the rocks. For instance, our investigation shows that samples (porphyry) with bigger grain size have lower modulus of elasticity. Also, based on the measurements using the point counting technique of grain size, inclusions, and matrix, samples with lower percentages of each component demonstrate lower value of indentation fracture resistance (**Table 5**). An experimental investigation by Huang and Wang [1985] on the impact of petrological characteristics, particularly of grain size to fracture toughness, suggest the dependence of these two parameters. However, due to the small size and unheated nature of studied samples compared to the thermally altered silcretes, our results should be considered with some caution. Further investigations involving larger number of samples will be required to assess whether the large grain size affected the mechanical properties of our porphyry or not.

Generally, the modulus of elasticity is proportional to the stiffness of the material. Domanski et al. [1994] states that the modulus of elasticity is an important measure of the suitability of materials for blade manufacture, because stiffness-controlled fracture propagation is basically responsible for blade detachment [10].

The mean values of K_{Ic} have a linear correlation with the E values meaning that samples with the lowest E values also present low K_{Ic} values and vice versa (see **Fig. 3 and 4**). The lower values of K_{Ic} imply less resistance against fracture suggesting that the material is easier to fracture and perhaps also to knap tools. Thus, our K_{Ic} suggest good knapping quality of

porphyry, despite observations to the contrary (see **subchapter 5.2** for discussion). Also, the modulus of elasticity and fracture resistance values of porphyry (see **Table 2**) can be compared to those of chert and silcrete obtained by Schmidt et al. [2019]. However, the meaning of K_{Ic} for the actual knapping properties observed during experimental knapping should be investigated in the future.

It is the first-time experiments such as this is being conducted to investigate the mechanical properties of *archaeological* porphyry. Our results offer a preliminary insight into the knapping quality of this rock. Further experimental lithic knapping or research on other mechanical properties (e.g., fracture strength) is necessary to examine the factors that affect the flaking of investigated samples. Similar research specifically targeting the evolution of mechanical properties of silica rocks upon heat treatment allow us to compare the K_{Ic} values with previously published data and verify the results. For instance, the fracture resistance values of chert and silcrete from Schmidt et al. [2019] study vary from 1.3 to 1.85 MPa $m^{1/2}$. The resistance values of our samples vary between 1.6 and 2.4 MPa $m^{1/2}$ and therefore support previous works.

5.2 Implications for stone tool knapping

Comparing the results of the experimental works with the stone tool knapping of various Upper Palaeolithic sites shows a pattern that allows us to preliminarily discuss the effect of raw material quality to the stone tool knapping technology. Although homogeneous, isotropic, fine-grained, and silica rich rocks were normally preferred for the manufacture of stone tools, evidence of the utilisation of locally available, and primarily considered of "lower" quality materials such as porphyry is known in Central Asian stratified Palaeolithic sites of Rahat and Maibulaq in Kazakhstan [60,62], and Kattasai-1 and Kattasai-2 in Uzbekistan [5,72]. We primarily concentrate our discussion on the quality of porphyry and the major techno-typological similarities between Palaeolithic industries that utilised it as a principal raw material.

Since porphyry was locally available as river pebbles, the large proportion of lithics knapped at the aforementioned sites were on this material. The use of higher quality exogeneous raw materials such as chert is common, but in much smaller quantities. Based on comprehensive literature review, we observed the presence of Levallois or Levallois-like technologies in all sites that utilise porphyry [5,60,62,72]. The lithic analysis of Kattasai-1 assemblage determined several schemes of reduction. The radial reduction of cores is predominant along with single and double platform parallel reduction [73]. Similar knapping technology was defined in both Maibulaq and Rahat. Besides the Middle Palaeolithic character of the Kattasai-1 and Upper Palaeolithic assemblages from Maibulaq and Rahat, technological analogies clearly demonstrate the link between the raw material quality and reduction strategies. We hypothesise that these similarities could be linked to the mechanical properties of porphyry. Additionally, our results suggest that porphyry has at least some properties that can be compared with those of chert. However, compared to chert porphyry is a coarse grained volcanic rock with a siliceous matrix (see **Table 4**). In coarser grained rocks the fracture propagates as both transgranular and intergranular cracks, thus consuming less energy to detach a flake of a desired morphology [74]. Moreover, the presence of bladelets knapped on both porphyry and shale from Maibulaq demonstrates the suitability for the production of smaller tools. The fracture resistance, elasticity, and hardness values obtained from our mechanical tests also attest the suitability for the knapping of blades and bladelets. This data allows us to hypothesise that hominins that once occupied these sites had to adapt the stone knapping technology to the quality and availability of resources. In addition to the grain size of porphyry, we observed that cobbles currently available in the Maibulaq stream bed today tend to have higher incidence of large phenocrysts than those encountered in the archaeological collection. Exploring this pattern with a sample of both raw material and archaeological stone tools could potentially indicate that prehistoric communities at Maibulaq deliberately selected rocks with smaller phenocrysts for knapping, but this must be tested with further analyses of raw materials and archaeological stone tools.

Conclusions

The present study for the first time tests fracture resistance, hardness, and modulus of elasticity of unheated samples of archaeological lithics and rocks from various Upper Palaeolithic sites in Kazakhstan. It also offers first insight into the quality of archaeological porphyry which was used as a principal raw material at various Upper Palaeolithic sites in the foothill and piedmont zones of the IAMC. We conclude that raw materials that were previously thought to be of lower quality (e.g., porphyry), in fact have some mechanical properties similar to those of chert. The prehistoric knappers that inhabited the northern foothills of the IAMC adapted the lithic reduction schemes to the quality of available raw material such as porphyry. The presence of levallois or levallois-like technology that can be observed from several Upper Palaeolithic sites that utilise porphyry as a main raw material support this hypothesis.

This work highlights the importance of the mechanical properties of chert, shale, and porphyry and its effect to the production of stone tools. Our findings show that materials that are expected to be difficult to knap based on visual criteria, objectively may have some good fracture properties. Hominins were most likely aware of this, and this kind of analysis shows that we have to base our conclusions on objective criteria rather than subjective ones. The porphyry case is a surprise because it is rarely used for knapping, and mainly unfamiliar to archaeologists[illegible] However, these kinds of counterintuitive results are also likely for some materials that *are* familiar to archaeologists, such as different types of cherts, basalts, and others.

Further investigations involving larger number and various types of raw materials and archaeological lithics are necessary to study and eventually build a catalogue of the mechanical properties of different raw materials from Kazakhstan. Our preliminary data can serve as a baseline for future quantitative and experimental investigations.

Acknowledgements

We would like to thank Dean Mendigul Nogaibaeva, Prof. Gani Omarov and Dr. Rinat Zhumatayev (Department of Archaeology, Ethnology and Museology, Faculty of History, Ethnology and Archaeology, Al Farabi Kazakhstan National University) for their support of this work. A. Namen would like to thank Prof. Dr. Christopher Miller for his support of this research, and access to the equipment of the Geoarchaeology laboratory at the Institute for Archaeological Sciences, in the University of Tübingen. The current study was performed within the PALAEOSILKROAD project and all field research was conducted under license No. 15008746 (12.05.2015) of the National Museum of the Republic of Kazakhstan based on the collaboration protocol between the Eberhard-Karls University of Tübingen and the National Museum. This project has received funding from the European Research Council (ERC) under the European Union's Horizon 2020 research and innovation programme (grant agreement n° 714842; PALAEOSILKROAD project). We acknowledge support by the Open Access Publishing Fund of the University of Tübingen.

Bibliography

1. Andrefsky W. The geological occurrence of lithic material and stone tool production strategies. Geoarchaeology. 1994;9(5):375–91.

2. Brantingham PJ, Olsen JW, Rech JA, Krivoshapkin AI. Raw Material Quality and Prepared Core Technologies in Northeast Asia. Journal of Archaeological Science. 2000 Mar;27(3):255–71.

3. Kuhn SL. Upper Paleolithic raw material economies at Üçağızlı cave, Turkey. Journal of Anthropological Archaeology. 2004 Dec;23(4):431–48.

4. Braun DR, Plummer T, Ferraro JV, Ditchfield P, Bishop LC. Raw material quality and Oldowan hominin toolstone preferences: evidence from Kanjera South, Kenya. Journal of Archaeological Science. 2009 Jul;36(7):1605–14.

5. Kot M, Pavlenok K, Krajcarz MT, Pavlenok G, Shnaider S, Khudjanazarov M, et al. Raw material procurement as a crucial factor determining knapping technology in the Katta Sai complex of Middle Palaeolithic sites in the western Tian Shan piedmonts of Uzbekistan. Quaternary International. 2020 Apr;S1040618220301580.

6. Shimelmitz R, Kuhn SL, Weinstein-Evron M. The evolution of raw material procurement strategies: A view from the deep sequence of Tabun Cave, Israel. Journal of Human Evolution. 2020 Jun;143:102787.

7. Goldman-Neuman T, Hovers E. Raw material selectivity in Late Pliocene Oldowan sites in the Makaamitalu Basin, Hadar, Ethiopia. Journal of Human Evolution. 2012 Mar;62(3):353–66.

8. Crabtree DE. Notes on Experiments in Flintknapping 3: The Flintknapper's Raw Materials. Tehiwa. 1967;10(1):8–25.

9. Cotterell B, Kamminga J, Dickson FP. The essential mechanics of conchoidal flaking. Int J Fract. 1985 Dec 1;29(4):205–21.

10. Cotterell B, Kamminga J. The Formation of Flakes. American Antiquity. 1987;52(4):675–708.

11. Collins S. Experimental investigations into edge performance and its implications for stone artefact reduction modelling. Journal of Archaeological Science. 2008;7.

12. Manninen MA. The effect of raw material properties on flake and flake-tool dimensions: A comparison between quartz and chert. Quaternary International. 2016 Dec;424:24–31.

13. Dogandžić T, Abdolazadeh A, Leader G, Li L, McPherron SP, Tennie C, et al. The results of lithic experiments performed on glass cores are applicable to other raw materials. Archaeol Anthropol Sci. 2020 Feb;12(2):44.

14. Marreiros J, Pereira T, Iovita R. Controlled experiments in lithic technology and function. Archaeol Anthropol Sci. 2020 Jun;12(6):110, s12520-020-01059–5.

15. McPherron SP, Abdolahzadeh A, Archer W, Chan A, Djakovic I, Dogandžić T, et al. Introducing platform surface interior angle (PSIA) and its role in flake formation, size and shape. PLOS ONE. 2020 Nov 18;15(11):e0241714.

16. Goodman ME. The Physical Properties of Stone Tool Materials. Am antiq. 1944 Apr;9(4):415–33.

17. Moník M, Hadraba H. Mechanical characterization of raw material quality and its implication for Early Upper Palaeolithic Moravia. Quaternary International. 2016 Dec;425:425–36.

18. Lerner H, Du X, Costopoulos A, Ostoja-Starzewski M. Lithic raw material physical properties and use-wear accrual. Journal of Archaeological Science. 2007;34(5):711–22.

19. Schmidt P, Fröhlich F. Temperature dependent crystallographic transformations in chalcedony, SiO2, assessed in mid infrared spectroscopy. Spectrochimica Acta Part A: Molecular and Biomolecular Spectroscopy. 2011 May;78(5):1476–81.

20. Domanski M, Webb JA, Boland J. Mechanical properties of stone artefact materials and the effect of heat treatment. Archaeometry. 1994 Aug;36(2):177–208.

21. Domanski M, Webb J. A Review of Heat Treatment Research. Lithic Technology. 2007 Jan;32(2):153–94.

22. Schmidt P, Sanchez OS, Kind C-J. Stone heat treatment in the Early Mesolithic of southwestern Germany: Interpretation and identification. PLOS ONE. 2017 Dec 6;12(12):e0188576.

23. Schmidt P, Buck G, Berthold C, Lauer C, Nickel KG. The mechanical properties of heat-treated rocks: a comparison between chert and silcrete. Archaeol Anthropol Sci. 2019 Jun;11(6):2489–506.

24. Mraz V, Fisch M, Eren MI, Lovejoy CO, Buchanan B. Thermal engineering of stone increased prehistoric toolmaking skill. Sci Rep. 2019 Dec;9(1):14591.

25. Schmidt P. When was silcrete heat treatment invented in South Africa? 2020;10.

26. Moník M, Nerudová Z, Schnabl P. Investigation of heat-treated artefacts from Pleistocene sites. Journal of Archaeological Science: Reports. 2021 Jun;37:102920.

27. Frachetti MD. Multiregional Emergence of Mobile Pastoralism and Nonuniform Institutional Complexity across Eurasia. Current Anthropology. 2012 Feb;53(1):2–38.

28. Aubekerov B, Gorbunov A. Quaternary permafrost and mountain glaciation in Kazakhstan. Permafrost and Periglacial Processes. 1999;10(1):65–80.

29. Allen MB, Alsop GI, Zhemchuzhnikov VG. Dome and basin refolding and transpressive inversion along the Karatau Fault System, southern Kazakstan. Journal of the Geological Society. 2001 Jan 1;158(1):83–95.

30. Buslov MM, Kokh DA, De Grave J. Mesozoic-Cenozoic tectonics and geodynamics of Altai, Tien Shan, and Northern Kazakhstan, from apatite fission-track data. Russian Geology and Geophysics. 2008 Sep 1;49(9):648–54.

31. Kirscher U, Zwing A, Alexeiev DV, Echtler HP, Bachtadse V. Paleomagnetism of Paleozoic sedimentary rocks from the Karatau Range, Southern Kazakhstan: Multiple remagnetization events correlate with phases of deformation. Journal of Geophysical Research: Solid Earth. 2013;118(8):3871–85.

32. Fitzsimmons KE, Iovita R, Sprafke T, Glantz M, Talamo S, Horton K, et al. A chronological framework connecting the early Upper Palaeolithic across the Central Asian piedmont. Journal of Human Evolution. 2017 Dec;113:107–26.

33. Khatsenovich AM, Rybin EP, Bazargur D, Marchenko DV, Kogai SA, Shevchenko TA, et al. Middle Palaeolithic human dispersal in Central Asia: new archaeological investigations in the Orkhon Valley, Mongolia. Antiquity [Internet]. 2019 Aug [cited 2020 Sep 4];93(370). Available from: https://www.cambridge.org/core/journals/antiquity/article/middle-palaeolithic-human-dispersal-in-central-asia-new-archaeological-investigations-in-the-orkhon-valley-mongolia/A046291999CC344BF7B6160DAFB8C054

34. Li, Vanwezer N, Boivin N, Gao X, Ott F, Petraglia M, et al. Heading north: Late Pleistocene environments and human dispersals in central and eastern Asia. PLOS ONE. 2019 May 29;14(5):e0216433.

35. Zwyns N, Paine CH, Tsedendorj B, Talamo S, Fitzsimmons KE, Gantumur A, et al. The northern route for human dispersal in Central and Northeast Asia: New evidence from the site of Tolbor-16, Mongolia. Sci Rep. 2019 Aug 13;9(1):1–10.

36. Iovita R, Varis A, Namen A, Cuthbertson P, Taimagambetov Z, Miller CE. In search of a Paleolithic Silk Road in Kazakhstan. Quaternary International. 2020 Sep;559:119–32.

37. Glantz MM. The History of Hominin Occupation of Central Asia in Review. In: Norton CJ, Braun DR, editors. Asian Paleoanthropology [Internet]. Dordrecht: Springer Netherlands; 2011 [cited 2020 Mar 31]. p. 101–12. (Vertebrate Paleobiology and Paleoanthropology). Available from: http://link.springer.com/10.1007/978-90-481-9094-2_8

38. Beeton TA, Glantz MM, Trainer AK, Temirbekov SS, Reich RM. The fundamental hominin niche in late Pleistocene Central Asia: a preliminary refugium model. Riddle B, editor. J Biogeogr. 2014 Jan;41(1):95–110.

39. Glantz M, Van Arsdale A, Temirbekov S, Beeton T. How to survive the glacial apocalypse: Hominin mobility strategies in late Pleistocene Central Asia. Quaternary International [Internet]. 2016 [cited 2016 Sep 15]; Available from: http://www.sciencedirect.com/science/article/pii/S1040618215302159

40. Medoev AG. Kamennyi vek Sary-Arka v svete noveishikh issledovanyi [Stone Age of Sary-Arka in the light of the latest research]. 1964;6(Izvestiya AN KazSSR):55–66.

41. Medoev AG. Geohronologiya paleolita Kazahstana. Alma Ata: Nauka; 1982. 64 p.

42. Alpysbaev Kh. O lokal'nykh razlichiyakh paleoliticheskikh kul'tur Srednei Azii i Kazakhstana [On local differences of the Paleolithic cultures of Central Asia and Kazakhstan]. In: Uspekhi sredneaziatskoi arkheologii. 2nd ed. Leningrad: Nauka; 1972. p. 5–7.

43. Alpysbaev Kh. Pamyatniki nizhnego paleolita yuzhnogo Kazakhstana [Lower Paleolithic sites of Southern Kazakhstan]. Alma-Ata: Nauka; 1979.

44. Alpysbaev Kh, Kostenko N. Stratigraficheskie usloviya nekotorykh paleoliticheskikh stoyanok Yuzhnogo Kazakhstana [Stratigraphic conditions of some Palaeolithic sites of southern Kazakhstan]. In: V glub' vekov (arkheologicheskii sbornik). Alma-Ata: Nauka KazSSR; 1974. p. 3–15.

45. Taimagambetov Zh. Shulbinskaya stoyanka [Shulbinka site]. Arkheologiya epokhi kamnya i metalla Sibiri Novosibirsk. 1983;161–7.

46. Taimagambetov Zh. Paleoliticheskaya stoyanka im. Ch. Valikhanova [The Ch. Valikhanova Paleolithic site]. Alma Ata: Nauka KazSSR; 1990.

47. Taimagambetov Zh. Periods in the development of Kazak palaeolithic industries: Their chronology and correlation with the industries in contiguous territories. East and West. 1997;47(1/4):351–61.

48. Derevianko AP, Petrin VT, Taimagambetov ZK, Zenin AN, Gladyshev SA. Paleoliticheskie kompleksy poverhnostnogo zaleganiya Mugodzharskih gor [Surface

Paleolithic complexes of the Mugodzharian mountains]. Problemy arxeologii, etnografii, antropologii Sibiri i sopredel'nyh teritoriy. 1999;5:50–5.

49. Derevianko AP, Petrin VT, Taimagambetov ŽK, Isabekov ZK, Rybalko RG, Otte M. Rannepaleoliticheskie mikroindustrial'nye compleksy v travertinakh yuzhnogo Kazakhstana [Lower Paleolithic microindustrial travertine complexes of Southern Kazakhstan]. Novosibirsk: Russian Academy of Sciences, Siberian Branch; 2000. (Izd-vo IAE SB RAS).

50. Derevianko AP. The lower paleolithic small tool industry in Eurasia: Migration or convergent evolution? Archaeology, Ethnology and Anthropology of Eurasia. 2006 Jul;25(1):2–32.

51. Artyukhova OA, Mamirov TB. Kamennyi vek Saryarki: evolutsiya kultur ot drevnepaleoliticheskikh okhotnikov do pervykh metallurgov v svete noveishikh issledovaniy [Stone Age of Saryarka: the evolution of cultures from ancient Paleolithic hunters to the first metallurgists in the light of the latest research]. Almaty: Institut arkheologii A. Kh. Margulana; 2014.

52. Shunkov M, Anoikin A, Taimagambetov Z, Pavlenok K, Kharevich V, Kozlikin M, et al. Ushbulak-1: new Initial Upper Palaeolithic evidence from Central Asia. Antiquity. 2017 Dec;91(360):e1.

53. Namen A, Aristeidis V, Cuthbertson P, Iovita R, Taimagambetov Z. Predvaritel'nye itogi razvedovatel'nykh rabot proekta PALAEOSILKROAD: mul'tidistsiplinarnyi podkhod v issledovaniyakh [Preliminary survey resuts of the PALAEOSILKROAD project: multidisciplinary approach]. In: Proceedings of the International scientific and practical conference 'Great Steppe in light of archaeological and interdisciplinary research'. Almaty: A. Kh. Margulan Institute of Archaeology; 2020. p. 60–9.

54. Namen A, Cuthbertson P, Varis A, Schmidt P, Taimagambetov Z, Iovita R. Preliminary results of the first lithic raw material survey in the piedmont zones of Kazakhstan [Internet]. SocArXiv; 2020 Sep [cited 2020 Dec 4]. Available from: https://osf.io/preprints/socarxiv/uztq6/

55. Hijmans R. GADM database of global administrative areas (version 2.0). 2012.

56. Jarvis A, Reuter HI, Nelson A, Guevara E. Hole-filled SRTM for the globe Version 4 [Internet]. 2008. Available from: http://srtm.csi.cgiar.org

57. Cuthbertson P, Ullmann T, Büdel C, Varis A, Namen A, Seltmann R, et al. Finding karstic caves and rockshelters in the Inner Asian mountain corridor using predictive modelling and field survey. PLOS ONE. 2021 Jan 20;16(1):e0245170.

58. Anoikin AA, Pavlenok GD, Kharevich VM, Taimagambetov ZK, Shalagina AV, Gladyshev SA, et al. Ushbulak - novaya mnogosloinaya stoyanka verkhnego paleolita na severo-vostoke Kazakhstana [Ushbulak - a new stratfied Upper Paleolithic site in Northeastern Kazakhstan]. Archaeology, Ethnography and Anthropology of Eurasia. 2019;47(4):16–29.

59. Taimagambetov Zh, Ozherelyev D. Izuchenie stratifitsirovannoy stoyanki Maibulak v Zhetisu v 2004-2007 g. [Studies at the stratified site of Maibulak in Zhetisu, 2004-2007]. Miras. 2008;1:70–86.

60. Taimagambetov Zh. Maibulak–First stratified Paleolithic site in Zhetysu (Kazakhstan). In: Derevianko AP, Shunkov MV, editors. Drevneishie migratsii cheloveka v Evrazii: materialy Mezhdunarodnogo simpoziuma, (6-12 sentiabria 2009 g, Makhachkala, Respublika Dagestan, Rossiia) [The earliest human migrations in Eurasia: proceedings of the International Symposium (Makhachkala, Dagestan Republic, Russia, September 6-12, 2009]. Novosibirsk: Institute of Archaeology and Ethnography Press; 2009. p. 301–9.

61. Alpysbaev Kh. Novye paleoliticheskie mestonakhozhdeniya v basseine rek Arystandy - Buryltai (Borolodai) v Yuzhnom Kazakhstane [New Paleolithic Locations in the Arystandy - Buryltai (Boroldai) River Basin in Southern Kazakhstan]. In: Novye materialy po arkheologii i etnografii Kazakhstana. Alma-Ata: Izdat. A. N. KazSSR; 1961. p. 3–20.

62. Ozherelyev DV, Dzhasybaev EA, Mamirov TB. Pervye dannye o stratigrafii i kul’turnoĭ atribucii mnogosloĭnoĭ stojanki verhnego paleolita Rahat (Jugo-Vostochnyĭ Kazahstan) [First data on the stratigraphy and cultural attribution of the Upper Paleolithic site of Rakhat (Southeast Kazakhstan)]. Kratkie soobshcheniya instituta arkheologii RAN Moskva. 2019;(254):57–70.

63. Niihara K, Morena R, Hasselman DPH. Evaluation ofK Ic of brittle solids by the indentation method with low crack-to-indent ratios. J Mater Sci Lett. 1982 Jan;1(1):13–6.

64. Danzer R, Lube T, Rasche S. On the Development of Experimental Methods for the Determination of Fracture Mechanical Parameters of Ceramics. In: Hütter G, Zybell L, editors. Recent Trends in Fracture and Damage Mechanics. Cham: Springer International Publishing; 2016. p. 197–214.

65. Ben Ghorbal G, Tricoteaux A, Thuault A, Louis G, Chicot D. Comparison of conventional Knoop and Vickers hardness of ceramic materials. Journal of the European Ceramic Society. 2017 Jun;37(6):2531–5.

66. Marshall DB, Noma T, Evans AG. A Simple Method for Determining Elastic-Modulus-to-Hardness Ratios using Knoop Indentation Measurements. J American Ceramic Society. 1982 Oct;65(10):c175–6.

67. Lawn R, Wilshaw TR. Fracture of brittle solids. Cambridge: Cambridge University Press; 1975. 204 p.

68. Courty, Goldberg P, Macphail R. Soils and Micromorphology in Archaeology. Cambridge University Press; 1989. 386 p.

69. Stoops G. Guidlines for analysis and description of soil and regolith thin sections. Madison: Soil Science Society of America; 2003. 181 p.

70. Harwood G. Microscopic techniques: II. principles of sedimentary petrography. In: Techniques in sedimentology. Oxford: Blackwell Scientific; 1988. p. 73–108.

71. Huang J-A, Wang S. An experimental investigation concerning the comprehensive fracture toughness of some brittle rocks. International Journal of Rock Mechanics and Mining Sciences & Geomechanics Abstracts. 1985 Apr 1;22(2):99–104.

72. Krajcarz MT, Kot M, Pavlenok K, Fedorowicz S, Krajcarz M, Lazarev SYu, et al. Middle Paleolithic sites of Katta Sai in western Tian Shan piedmont, Central Asiatic loess zone: Geoarchaeological investigation of the site formation and the integrity of the lithic assemblages. Quaternary International [Internet]. 2015 [cited 2015 Sep 2]; Available from: http://www.sciencedirect.com/science/article/pii/S1040618215007454

73. Pavlenok K, Kot M, Pavlenok G, Krajcarz MT, Khudjanazarov M, Leloch M, et al. Middle Paleolithic technological diversity during MIS 3 in the Western Tian Shan piedmonts: Example of the Katta Sai 1 open -air loess site. Archaeological Research in Asia. 2021 Feb;100262.

74. Mardon D, Kronenberg AK, Handin J, Friedman M, Russell JE. Mechanisms of fracture propagation in experimentally extended Sioux quartzite. Tectonophysics. 1990;182:259–78.

Figure 1

Click here to access/download;Figure;Fig. 1..tif

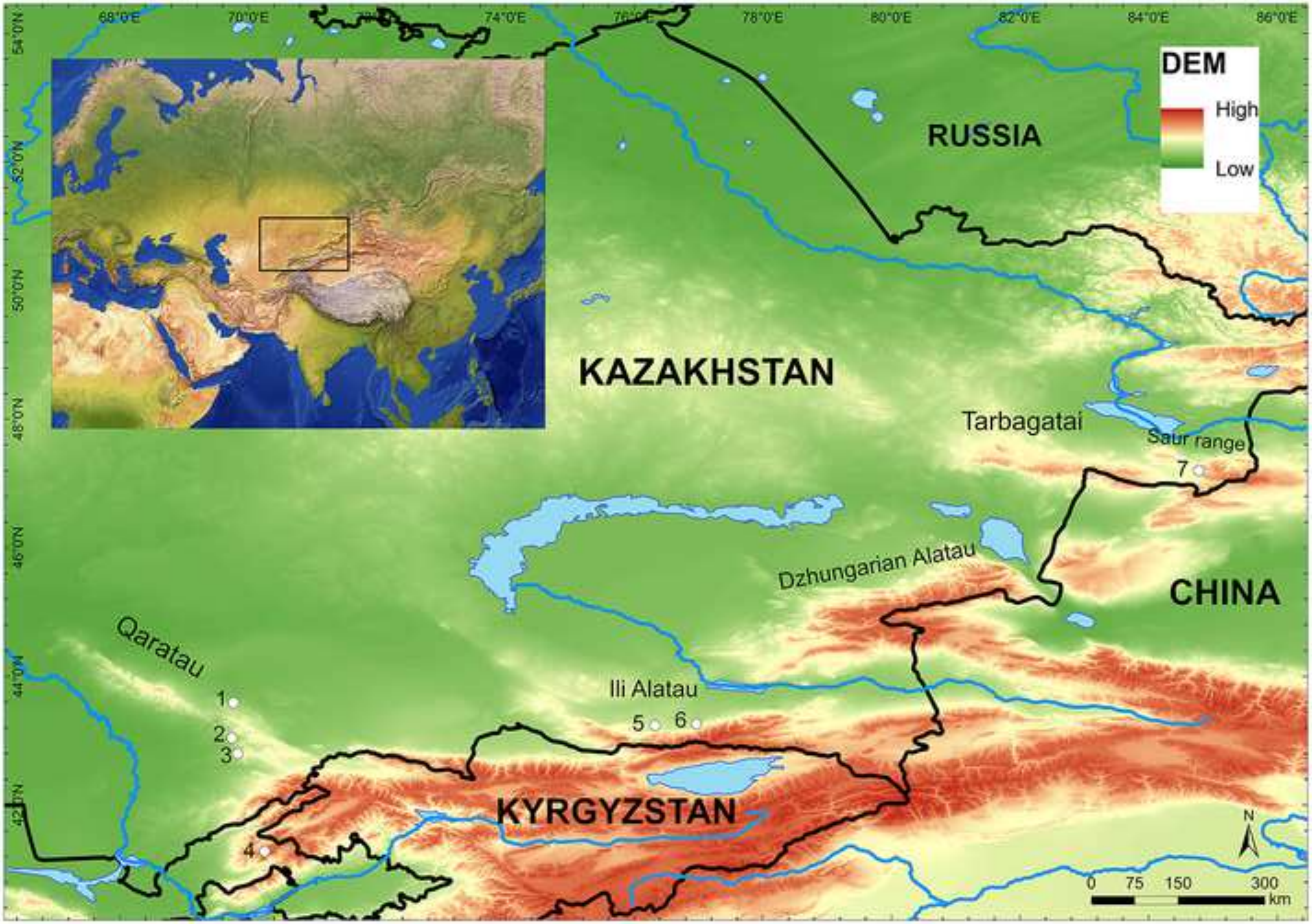

Figure 2

Click here to access/download;Figure;Fig. 2..tif

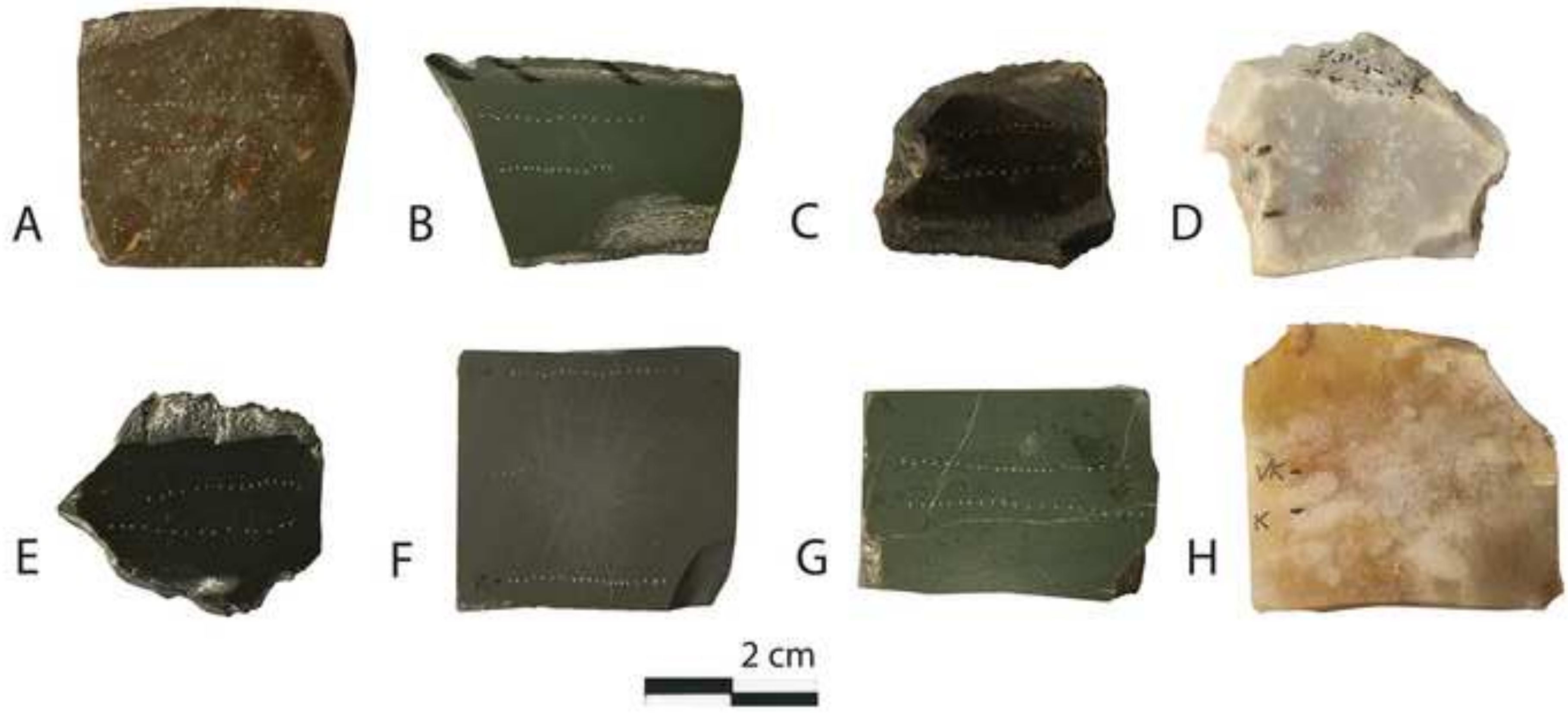

Figure 3

Click here to access/download;Figure;Fig. 3..tif

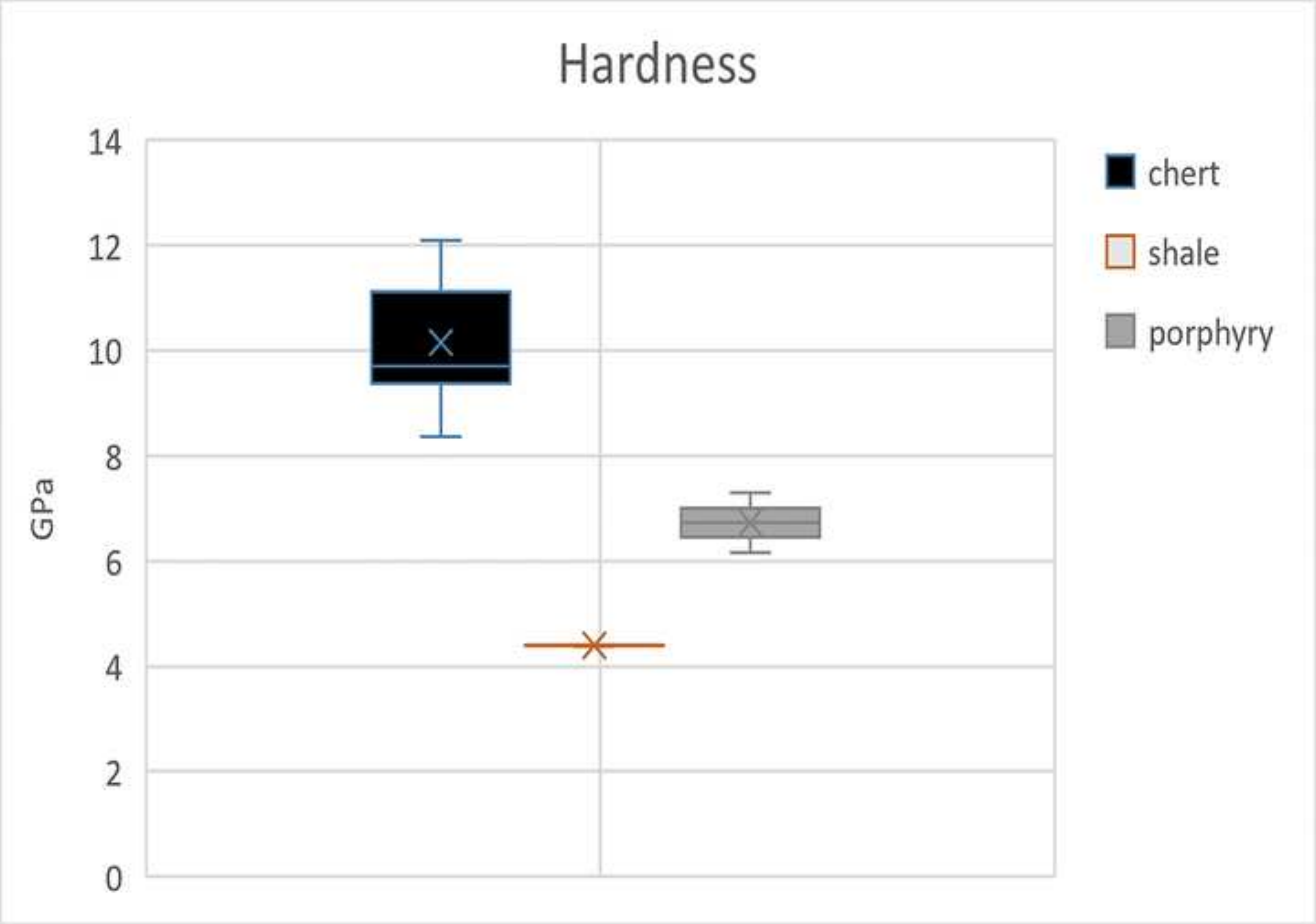

Figure 4

Click here to access/download;Figure;Fig. 4..tif

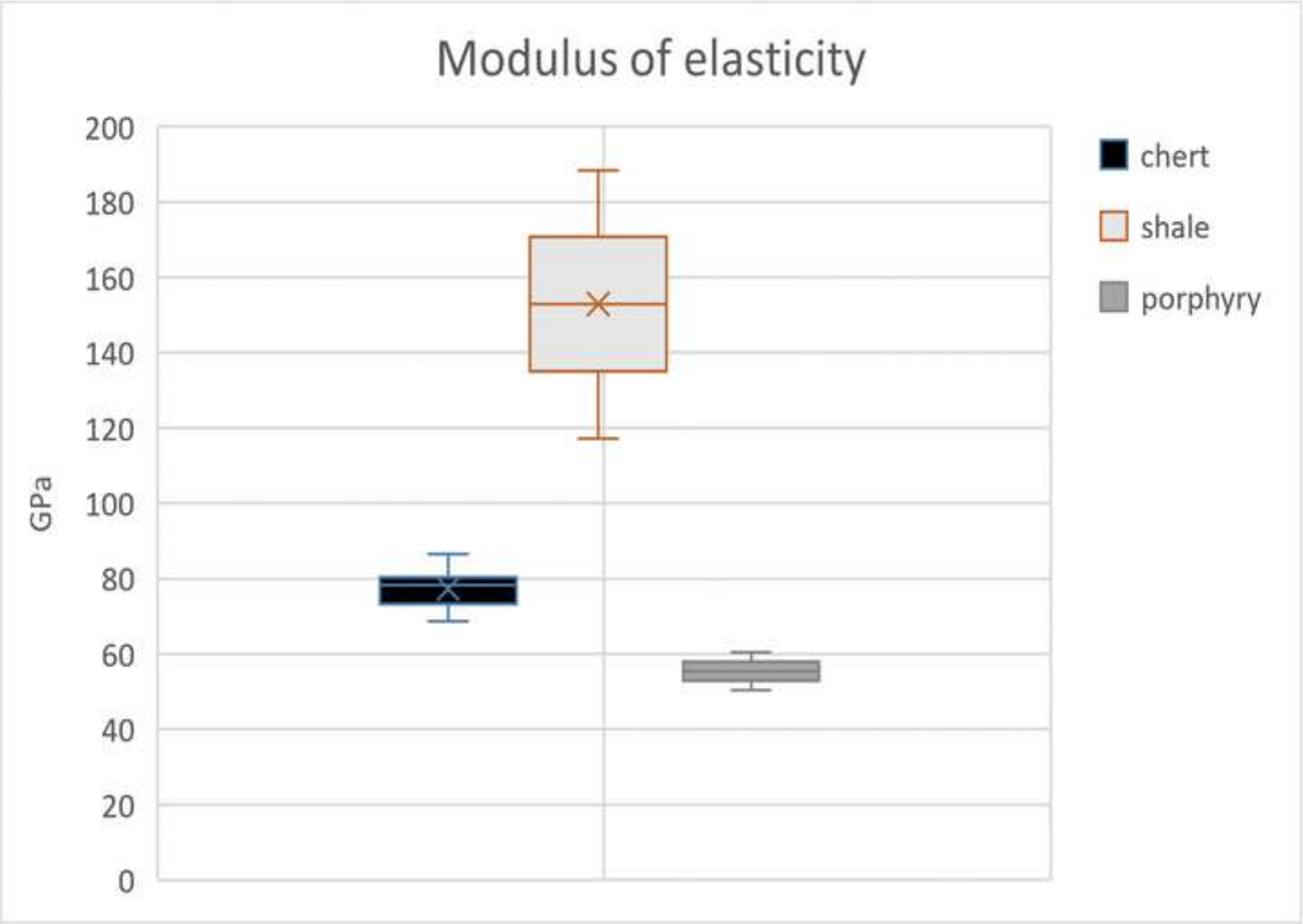

Figure 5

Click here to access/download;Figure;Fig. 5..tif

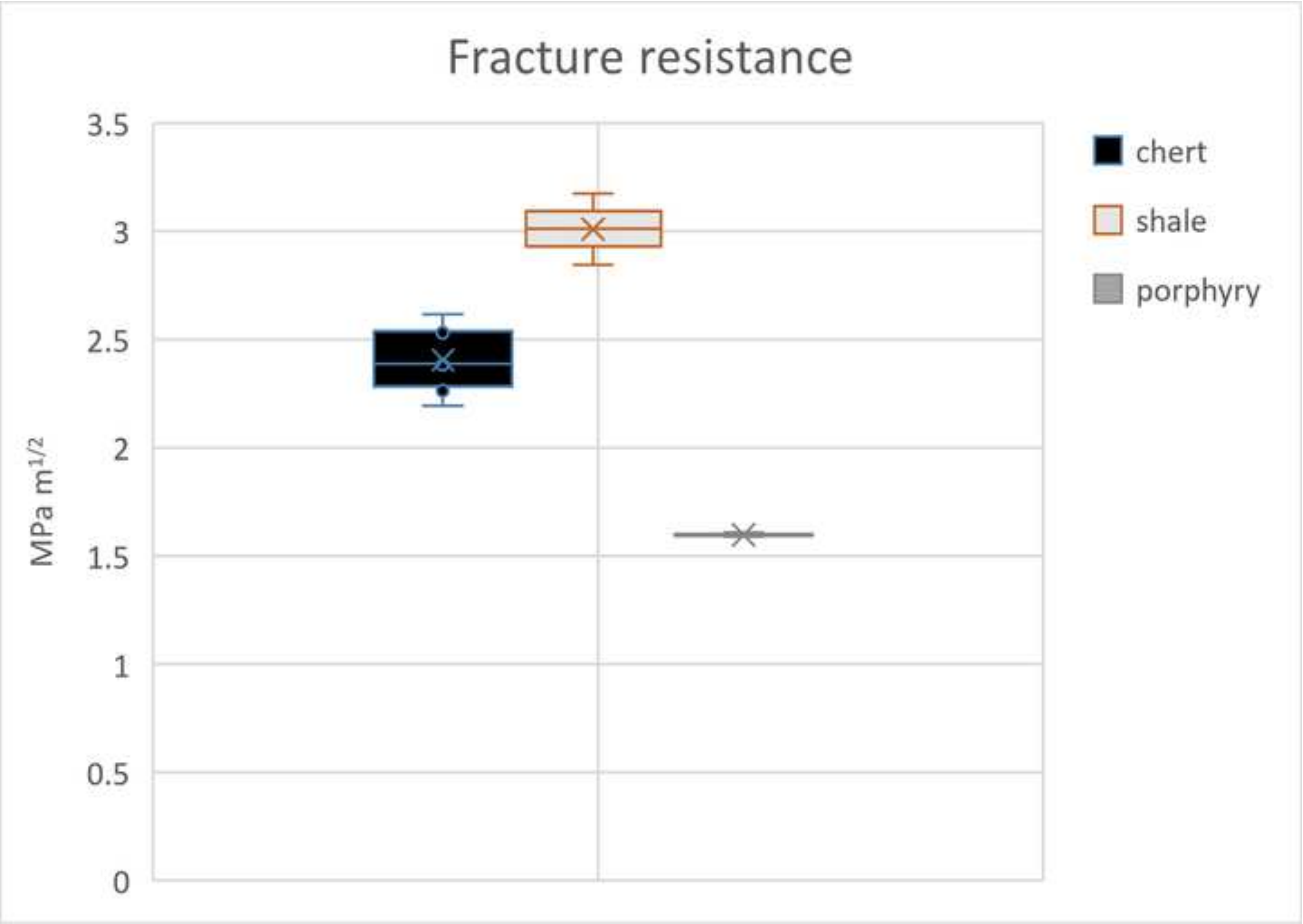

Figure 6

Click here to access/download;Figure;Fig. 6..tif

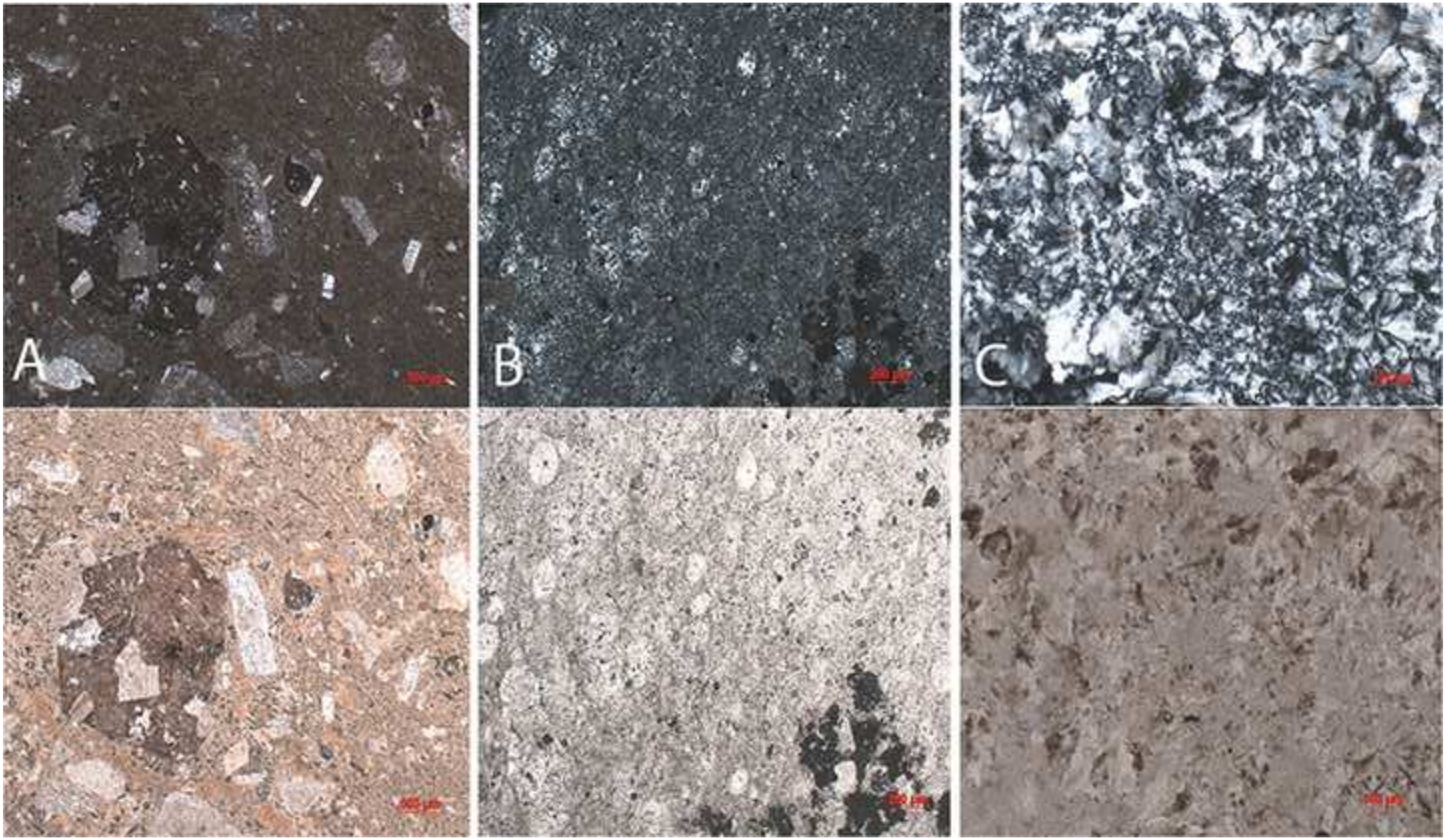

Figure 7

Click here to access/download;Figure;Fig. 7..tif

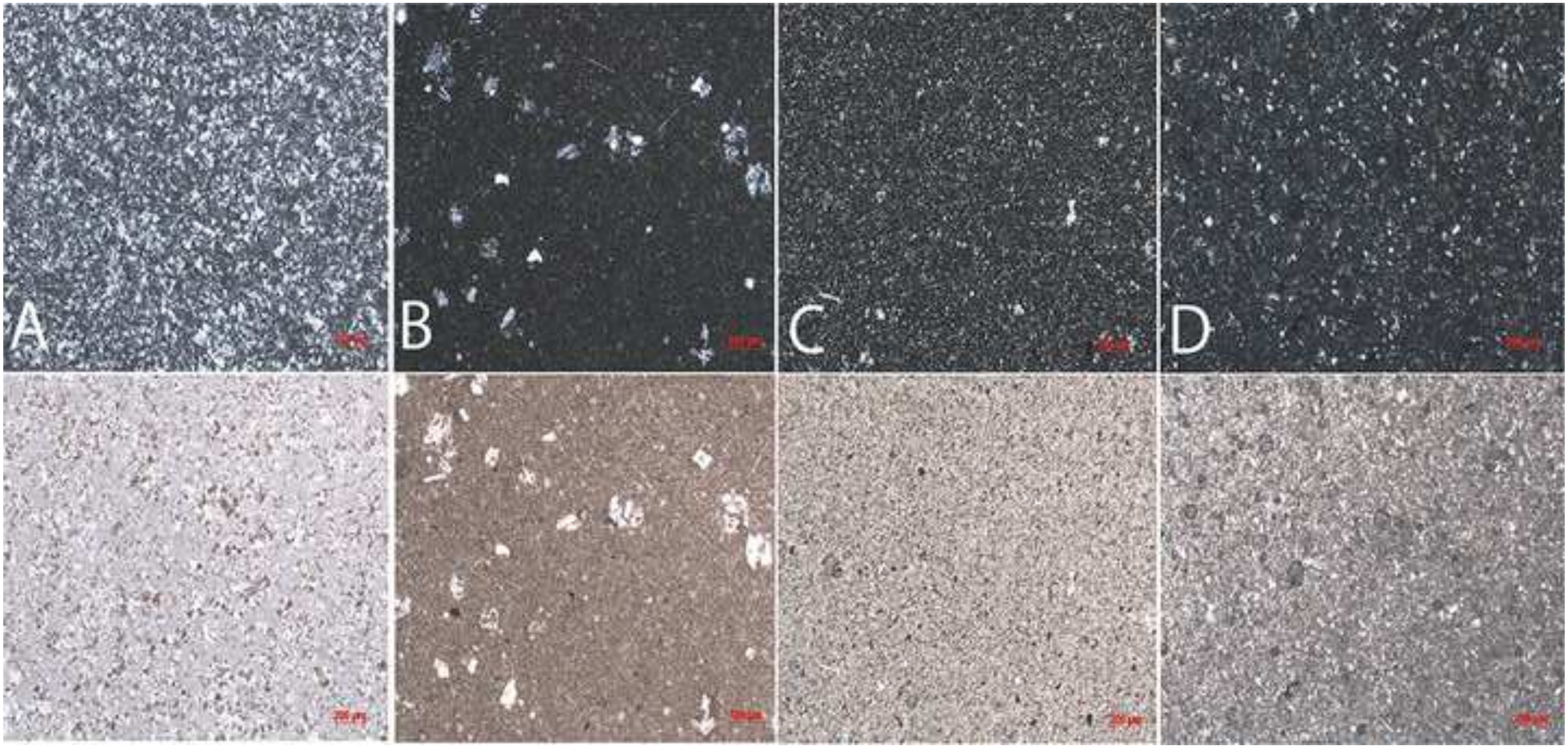

Supplement: Attachment — Submitted filename: PONE-D-21-32866_rev.pdf [file pone.0265640.s001.pdf]
