# Supplementary material for: Mechanical properties of lithic raw materials from Kazakhstan: Comparing chert, shale, and porphyry
Source: PLoS One. 2022 Apr 22;17(4):e0265640. doi: 10.1371/journal.pone.0265640 (PMC9033281; doi:10.1371/journal.pone.0265640)
Supplement: Attachment — Submitted filename: Response to Reviewers.docx [file pone.0265640.s003.docx]

Dear Dr. Spinapolice,

Many thanks for your work in managing this process, and for considering our manuscript for publication. In the revised cover letter, we address your questions regarding the possibility of copyrighted satellite imagery in our figures (our figures do not contain satellite imagery, so there is no copyright issue in this regard).

We greatly appreciate of the insightful comments on our manuscript from two anonymous reviewers. We respond to their comments in detail below.

Many thanks.

Dear Reviewer 1,

Thank you for your valuable work, the time you have spent on doing the review, and the comments you provided on our manuscript, especially your recommendations for clarity in phrasing and additional archaeological information. We appreciate your expertise and your constructive suggestions, which have helped us improve the quality of the paper.

We have revised the manuscript and addressed the issues indicated in your report. Changes are tracked in the revised version of the manuscript. Additionally, please find a point-to-point response to your questions/comments below.

Thanks again and kind regards.

**# Reviewer 1**

**The paper entitled “Mechanical properties of lithic raw materials from Kazakhstan: comparing chert, shale, and porphyry” by Abay Namen and colleagues aim to test some mechanical properties of raw materials from Kazakhstan in a comparative perspective, including petrographic studies as well. The main objectives of the study are: 1- to test geological and archaeological samples to determine their mechanical properties, and 2- to preliminarily assess how these properties affect the knapping technology. The paper focuses on an interesting topic: the importance of mechanical properties of raw materials and its implication for the comprehension of hominin behavior and lithic technology. Especially in the area of Kazakhstan, where the study has been settled, and in general for Central Asia. Thus, this is a great contribution to the knowledge of the aforementioned area.
The manuscript is well written and structured in clear sections, with many tables, which are very welcomed to understand the methodology of analysis. The data are available, and most of my comments are detailed in the attached pdf. The research background is well illustrated, as well as figures.**

**I think the experiments and test the authors used are appropriate to the research questions and objectives, and there are enough data to make the study replicable. The paper offers a window to a wider potential research topic that needs to be improved. The results shown in the manuscript highlight the importance of these types of objective tests, which represent an excellent tool to understand and interpret the archaeological contexts and the behavioral aspects related to the groups that occupied them.**

**For this manuscript, I would recommend minor revisions before publication, based on the following points that require clarification:**

**1. More details should be given on the collection of samples, the criteria applied to the selection, and the archaeological contexts where they came from (see attached pdf. for comments).**

We have added these details in the revised version of the manuscript.

**2. The tables presented in the text are cited in a confused order (e.g. Tab 4 is mentioned before tabs 2 and 3).**

We have fixed this issue in the manuscript. Please, see the revised version.

**3. Captions to Figures 3, 4, and 5 can be improved. It would be helpful to name the graph the authors used to illustrate the results of the tests.**

Thank you for this remark. We have added additional information in the captions of Fig 2-5.

**Finally, I would recommend the revision of the manuscript by an English native speaker.**

The revised version of the manuscript has been proofread by a native English speaker.

____________________________________________________________________

Dear reviewer 2,

Thank you for your valuable work, the time you have spent on doing the review, and the comments you provided on our manuscript. We appreciate your expertise and your constructive suggestions, which have helped us to improve the quality of the paper.

We have revised the manuscript and addressed the issues indicated in your report. Changes are tracked in the revised version of the manuscript. As well, please find a point-to-point response to your questions/comments below.

Thanks again and kind regards.

**# Reviewer 2:**

**This paper presents engaging data on raw material from Kazakhstan Upper Paleolithic sites and proposes an interesting methodology to study their mechanical properties. The indentation tests were complemented by traditional petrographic studies, which offer a supportive tool to support their hypothesis, re-evaluating the importance of porphyry as the primary source of artefacts manufacture. The manuscript is interesting, as it adds some details to an appealing – yet little known – topic, namely the exploited raw materials and their properties in central Asia during Upper Palaeolithic. Although the complete set of geological samples is wished to be enlarged in future studies, the described data represent a good overview, despite the absence of a porphyry geological sample that could have perfectly completed the framework.**

**However, several parts in the paper should be updated and rephrased to make them meet the specific targets of the publication.**

**The author's hypotheses are quite convincing, but probably more analyses should be necessary in order to adequately and undoubtedly clarify the unanswered question and extend the lithoteque, and thus raw material circulation routes.**

**At various places, data are presented in a much too concise way (as for the test and archaeological sample description), while the procedural part is sometimes too detailed. I am attaching the manuscript with in-text comments and questions, and you will find below a list of minor comments.**

**My general and minor comments are discussed hereafter. See the pdf for detailed comments.**

**All the words or periods reported between "" indicate the proposed alternatives to the original text.**

**All comments and deletions are followed by side-comment. If you are not able to see them, let the editor know it and I'll provide another file.**

**To conclude, this is a significant and valuable contribution to the archaeology of the region and for the lithic studies, and I recommend its publication with minor-to-moderate revisions.**

**General points:**

**1. The 'archaeological' part of the manuscript is absent. More information related to the contexts, chronological framework, and reference sites from where the absolute dates come from is more than welcomed. I do not think this might be considered a big issue – but on the other hand, it is true that the 'archaeological' descriptions of the investigated specimens are way too short.**

This is true. We have improved missing information concerning the chronology and context of the archaeological sites. They are also cited appropriately for further details.

**2. A minor problem is represented by the too "simplistic" lexicon that makes it look like an essay. I suggest a general revision from a native English speaker or a language polishing with special attention for syntax and words repetition. Also, the verbs concordance needs some attention.**

Thank you for your remark. The revised version of the manuscript has been proofread by a native English speaker.

**3. Lastly, there are some concepts repeated throughout the text. Try to delate these parts and substitute them with a brief explanation of why you chose specific approaches and what they consisted of.**

We have fixed these issues throughout the manuscript according to your side-comments in the PDF file.

**Minor comments:
· Citation of some page numbers in the Bibliography must be standardized.**

This issue was solved.

**· The illustration section should be slightly modified, adding details to the pictures of the sampled areas. You will find detailed requests in the pdf.**

Thank you for the remarks. We have incorporated these changes in the revised version of the manuscript.
